# (*Z*)-*N*-{3-[1-(4-Chloro­phen­yl)eth­yl]thia­zolidin-2-yl­idene}cyanamide

**DOI:** 10.1107/S1600536810029879

**Published:** 2010-07-31

**Authors:** Hong-xin Li, Xiao-jun Zhang, Liang-zhong Xu

**Affiliations:** a College of Chemistry and Molecular Engineering, Qingdao University of Science and Technology, Qingdao 266042, People’s Republic of China

## Abstract

The title compound, C_12_H_12_ClN_3_S, features a thia­zolyl ring having an envelope conformation with the –CH_2_– group bonded to the S atom forming the flap. The C=N double bond has a *Z* configuration. The crystal structure shows inter­molecular C—H⋯S hydrogen bonds.

## Related literature

For the biological activity of thia­zole componds, see: Hense *et al.* (2002 ▶). For a related structure, see: Cunico *et al.* (2007 ▶). For the synthesis, see: Jeschke *et al.* (2002 ▶).
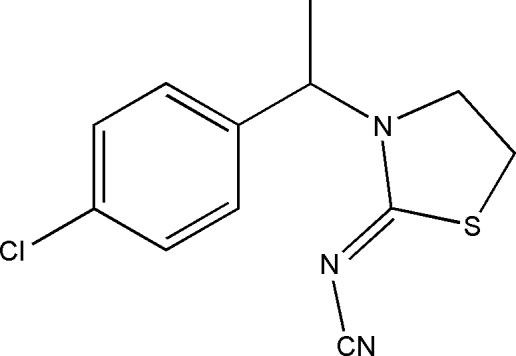


## Experimental

### 

#### Crystal data

C_12_H_12_ClN_3_S
*M* *_r_* = 265.76
Orthorhombic, 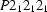

*a* = 5.8850 (12) Å
*b* = 7.5965 (15) Å
*c* = 28.273 (6) Å
*V* = 1264.0 (4) Å^3^
*Z* = 4
Mo *K*α radiation
μ = 0.45 mm^−1^
*T* = 113 K
0.14 × 0.12 × 0.10 mm

#### Data collection

Rigaku Saturn diffractometer
Absorption correction: multi-scan (*CrystalClear*; Rigaku, 2005 ▶) *T*_min_ = 0.940, *T*_max_ = 0.957
9219 measured reflections
3019 independent reflections
2640 reflections with *I* > 2σ(*I*)
*R*_int_ = 0.062

#### Refinement

*R*[*F* ^2^ > 2σ(*F* ^2^)] = 0.046
*wR*(*F* ^2^) = 0.113
*S* = 1.06
3019 reflections
155 parameters
H-atom parameters constrained
Δρ_max_ = 0.38 e Å^−3^
Δρ_min_ = −0.34 e Å^−3^
Absolute structure: Flack (1983 ▶), 6200 Friedel pairs
Flack parameter: −0.09 (9)

### 

Data collection: *CrystalClear* (Rigaku, 2005 ▶); cell refinement: *CrystalClear*; data reduction: *CrystalClear*; program(s) used to solve structure: *SHELXS97* (Sheldrick, 2008 ▶); program(s) used to refine structure: *SHELXL97* (Sheldrick, 2008 ▶); molecular graphics: *SHELXTL* (Sheldrick, 2008 ▶); software used to prepare material for publication: *SHELXTL*.

## Supplementary Material

Crystal structure: contains datablocks I, global. DOI: 10.1107/S1600536810029879/ng5002sup1.cif

Structure factors: contains datablocks I. DOI: 10.1107/S1600536810029879/ng5002Isup2.hkl

Additional supplementary materials:  crystallographic information; 3D view; checkCIF report

## Figures and Tables

**Table 1:** Hydrogen-bond geometry (Å, °)

*D*—H⋯*A*	*D*—H	H⋯*A*	*D*⋯*A*	*D*—H⋯*A*
C10—H10*A*⋯S1^i^	0.97	2.87	3.799 (4)	160

Symmetry code: (i) 
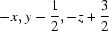
.

**Fig. 1.**
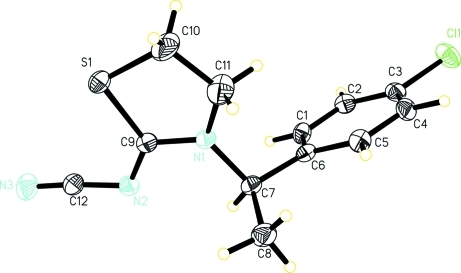
The molecular structure of (I), with displacement ellipsoids drawn at the 40% probability level.

## supplementary crystallographic information

### Comment

Recently, compounds containing the 2-(thiazolidin-2-ylidene)malononitrile group have attracted much interest because its containing a thiazole ring system are well known as efficient insecticide in pesticides, and have good plant-growth regulatory activity for a wide variety of crops *e.g.* thiacloprid (Hense *et al.*, 2002). A new compound, (I), which containing thiazole ring has higher insecticide activity. We report here the crystal structure and synthesis of (I).

In (I) (Fig. 1), the bond lengths and angles are normal and in a agreement with those common to a previously reported structure (Cunico, *et al.*, 2007). The thiazole ring is in and envelope conformation with the –CH_2_– group bonded to the S atom forming the flap. The carbon-nitrogen double bond of the molecule is *trans*. Flack *x* parameter = -0.0865 (with e.s.d. 0.0872) (Flack, 1983). The crystal structure involves C—H···S intermolecular hydrogen bonds.

### Experimental

(*Z*)-(thiazolidin-2-ylideneamino)formonitrile 1.27 g (10.0 mmol) and potassium carbonate (10.0 mmol) were dissolved in *N*,*N*-dimethylformamide(DMF) (15 ml) which was stirred 0.5 h at room temperature. Then 1-chloro-4-(1-chloroethyl)benzene 1.75 g (10.0 mmol) was added dropwising within 2 h at 283 K. The mixture was stirred for 8 h at 428 K. Upon cooling at room temperature. Then water (20 ml) was added. The mixture was extracted with CH_2_Cl_2_ (15 ml) and the organic layer was washed with water and dried over anhydrous sodium sulfate. The excess CH_2_Cl_2_ was removed on a water vacuum pump to obtain the oily product. Crystallized from methanol to afford the title compound 2.0 g (76% yield) (Jeschke, *et al.*, 2002.). Single crystals suitable for X-ray measurement were obtained by recrystallization from the mixture of acetone and methanol at room temperature.

### Refinement

All C-bound H atoms were placed in calculated positions, with C—H = 0.93–0.98 Å, and included in the final cycles of refinement using a riding model, with *U*_iso_(H) = 1.2*U*_eq_(C) for the aryl and 1.5 *U*_eq_(C) for methyl H atoms. Friedel pairs = 6200.

### Crystal data

**Table:**

C_12_H_12_ClN_3_S	*F*(000) = 552
*M**_r_* = 265.76	*D*_x_ = 1.397 Mg m^−^^3^
Orthorhombic, *P*2_1_2_1_2_1_	Mo *K*α radiation, λ = 0.71073 Å
Hall symbol: P 2ac 2ab	Cell parameters from 193 reflections
*a* = 5.8850 (12) Å	θ = 1.4–27.9°
*b* = 7.5965 (15) Å	µ = 0.45 mm^−^^1^
*c* = 28.273 (6) Å	*T* = 113 K
*V* = 1264.0 (4) Å^3^	Block, white
*Z* = 4	0.14 × 0.12 × 0.10 mm

### Data collection

**Table:**

Rigaku Saturn diffractometer	3019 independent reflections
Radiation source: rotating anode	2640 reflections with *I* > 2σ(*I*)
confocal	*R*_int_ = 0.062
ω scans	θ_max_ = 27.9°, θ_min_ = 1.4°
Absorption correction: multi-scan (*CrystalClear*; Rigaku, 2005)	*h* = −7→7
*T*_min_ = 0.940, *T*_max_ = 0.957	*k* = −9→8
9219 measured reflections	*l* = −29→37

### Refinement

**Table:**

Refinement on *F*^2^	Secondary atom site location: difference Fourier map
Least-squares matrix: full	Hydrogen site location: inferred from neighbouring sites
*R*[*F*^2^ > 2σ(*F*^2^)] = 0.046	H-atom parameters constrained
*wR*(*F*^2^) = 0.113	*w* = 1/[σ^2^(*F*_o_^2^) + (0.0534*P*)^2^ + 0.0295*P*] where *P* = (*F*_o_^2^ + 2*F*_c_^2^)/3
*S* = 1.06	(Δ/σ)_max_ = 0.001
3019 reflections	Δρ_max_ = 0.38 e Å^−^^3^
155 parameters	Δρ_min_ = −0.34 e Å^−^^3^
0 restraints	Absolute structure: Flack (1983), 6200 Friedel pairs
Primary atom site location: structure-invariant direct methods	

### Special details

**Table:**

Geometry. All e.s.d.'s (except the e.s.d. in the dihedral angle between two l.s. planes) are estimated using the full covariance matrix. The cell e.s.d.'s are taken into account individually in the estimation of e.s.d.'s in distances, angles and torsion angles; correlations between e.s.d.'s in cell parameters are only used when they are defined by crystal symmetry. An approximate (isotropic) treatment of cell e.s.d.'s is used for estimating e.s.d.'s involving l.s. planes.
Refinement. Refinement of *F*^2^ against ALL reflections. The weighted *R*-factor *wR* and goodness of fit *S* are based on *F*^2^, conventional *R*-factors *R* are based on *F*, with *F* set to zero for negative *F*^2^. The threshold expression of *F*^2^ > σ(*F*^2^) is used only for calculating *R*-factors(gt) *etc*. and is not relevant to the choice of reflections for refinement. *R*-factors based on *F*^2^ are statistically about twice as large as those based on *F*, and *R*- factors based on ALL data will be even larger.

### Fractional atomic coordinates and isotropic or equivalent isotropic displacement parameters (Å^2^)

**Table:**

	*x*	*y*	*z*	*U*_iso_*/*U*_eq_	
Cl1	0.31420 (16)	−0.62970 (7)	0.98241 (2)	0.0438 (2)	
S1	0.18444 (12)	0.16944 (8)	0.77090 (2)	0.03081 (17)	
N2	0.2773 (4)	0.3380 (3)	0.85392 (7)	0.0279 (4)	
C9	0.3024 (4)	0.1982 (3)	0.82718 (8)	0.0238 (5)	
C1	0.2990 (4)	−0.1331 (3)	0.93777 (8)	0.0267 (5)	
H1	0.2024	−0.0368	0.9407	0.032*	
N1	0.4250 (4)	0.0594 (2)	0.84129 (6)	0.0255 (5)	
C6	0.4992 (4)	−0.1158 (3)	0.91192 (8)	0.0242 (5)	
C7	0.5466 (4)	0.0572 (3)	0.88698 (8)	0.0257 (5)	
H7	0.4806	0.1507	0.9065	0.031*	
C8	0.7950 (5)	0.1029 (4)	0.87970 (9)	0.0369 (6)	
H8A	0.8064	0.2125	0.8630	0.055*	
H8B	0.8672	0.0117	0.8616	0.055*	
H8C	0.8686	0.1135	0.9099	0.055*	
C12	0.1462 (5)	0.4672 (3)	0.83801 (8)	0.0294 (6)	
C2	0.2409 (4)	−0.2905 (3)	0.95914 (8)	0.0288 (5)	
H2	0.1055	−0.3011	0.9759	0.035*	
C4	0.5886 (5)	−0.4192 (3)	0.93042 (9)	0.0316 (6)	
H4	0.6860	−0.5152	0.9284	0.038*	
C3	0.3874 (5)	−0.4315 (3)	0.95515 (8)	0.0285 (5)	
N3	0.0347 (5)	0.5868 (3)	0.82737 (8)	0.0414 (6)	
C5	0.6432 (5)	−0.2610 (3)	0.90865 (8)	0.0292 (6)	
H5	0.7778	−0.2518	0.8916	0.035*	
C11	0.4588 (7)	−0.0759 (4)	0.80600 (9)	0.0479 (8)	
H11A	0.6166	−0.0769	0.7961	0.058*	
H11B	0.4235	−0.1901	0.8195	0.058*	
C10	0.3144 (8)	−0.0437 (4)	0.76555 (12)	0.0648 (12)	
H10A	0.1977	−0.1336	0.7638	0.078*	
H10B	0.4039	−0.0488	0.7368	0.078*	

### Atomic displacement parameters (Å^2^)

**Table:**

	*U*^11^	*U*^22^	*U*^33^	*U*^12^	*U*^13^	*U*^23^
Cl1	0.0702 (5)	0.0288 (3)	0.0324 (4)	−0.0048 (3)	0.0066 (4)	0.0021 (3)
S1	0.0384 (4)	0.0355 (3)	0.0185 (3)	−0.0011 (3)	−0.0028 (3)	0.0002 (2)
N2	0.0319 (11)	0.0266 (9)	0.0252 (11)	−0.0013 (9)	−0.0015 (9)	−0.0014 (8)
C9	0.0227 (11)	0.0302 (11)	0.0186 (11)	−0.0047 (10)	0.0030 (10)	0.0020 (8)
C1	0.0253 (12)	0.0304 (11)	0.0246 (12)	0.0062 (11)	0.0008 (10)	0.0000 (9)
N1	0.0305 (11)	0.0255 (10)	0.0204 (11)	0.0005 (9)	−0.0029 (9)	−0.0032 (8)
C6	0.0232 (11)	0.0293 (11)	0.0201 (12)	−0.0007 (9)	−0.0038 (9)	−0.0022 (9)
C7	0.0267 (12)	0.0289 (12)	0.0215 (13)	−0.0010 (10)	−0.0029 (10)	−0.0016 (9)
C8	0.0319 (14)	0.0419 (14)	0.0368 (16)	−0.0080 (13)	−0.0029 (12)	0.0067 (12)
C12	0.0371 (15)	0.0332 (12)	0.0179 (12)	−0.0011 (11)	0.0008 (10)	−0.0010 (9)
C2	0.0284 (13)	0.0332 (12)	0.0249 (13)	−0.0021 (10)	0.0014 (10)	−0.0010 (10)
C4	0.0376 (14)	0.0252 (11)	0.0319 (14)	0.0079 (11)	−0.0017 (11)	−0.0064 (10)
C3	0.0386 (14)	0.0260 (11)	0.0210 (13)	−0.0031 (11)	−0.0019 (10)	−0.0016 (10)
N3	0.0575 (17)	0.0394 (12)	0.0273 (13)	0.0087 (12)	−0.0025 (11)	−0.0006 (10)
C5	0.0296 (14)	0.0329 (11)	0.0252 (14)	0.0034 (11)	0.0024 (10)	−0.0026 (10)
C11	0.069 (2)	0.0489 (16)	0.0254 (15)	0.0203 (17)	−0.0048 (14)	−0.0132 (13)
C10	0.097 (3)	0.0508 (18)	0.046 (2)	0.027 (2)	−0.038 (2)	−0.0243 (14)

### Geometric parameters (Å, °)

**Table:**

Cl1—C3	1.746 (2)	C8—H8A	0.9600
S1—C9	1.750 (2)	C8—H8B	0.9600
S1—C10	1.797 (3)	C8—H8C	0.9600
N2—C9	1.311 (3)	C12—N3	1.161 (3)
N2—C12	1.327 (3)	C2—C3	1.380 (4)
C9—N1	1.338 (3)	C2—H2	0.9300
C1—C2	1.383 (3)	C4—C3	1.378 (4)
C1—C6	1.393 (3)	C4—C5	1.388 (3)
C1—H1	0.9300	C4—H4	0.9300
N1—C11	1.446 (3)	C5—H5	0.9300
N1—C7	1.477 (3)	C11—C10	1.446 (4)
C6—C5	1.394 (3)	C11—H11A	0.9700
C6—C7	1.517 (3)	C11—H11B	0.9700
C7—C8	1.516 (4)	C10—H10A	0.9700
C7—H7	0.9800	C10—H10B	0.9700
			
C9—S1—C10	91.16 (13)	N3—C12—N2	174.7 (3)
C9—N2—C12	118.0 (2)	C3—C2—C1	118.8 (2)
N2—C9—N1	121.8 (2)	C3—C2—H2	120.6
N2—C9—S1	125.49 (18)	C1—C2—H2	120.6
N1—C9—S1	112.73 (17)	C3—C4—C5	118.9 (2)
C2—C1—C6	121.3 (2)	C3—C4—H4	120.6
C2—C1—H1	119.3	C5—C4—H4	120.6
C6—C1—H1	119.3	C4—C3—C2	121.7 (2)
C9—N1—C11	115.4 (2)	C4—C3—Cl1	119.64 (19)
C9—N1—C7	122.06 (19)	C2—C3—Cl1	118.6 (2)
C11—N1—C7	121.9 (2)	C4—C5—C6	121.0 (2)
C1—C6—C5	118.3 (2)	C4—C5—H5	119.5
C1—C6—C7	118.8 (2)	C6—C5—H5	119.5
C5—C6—C7	122.9 (2)	C10—C11—N1	110.1 (2)
N1—C7—C8	110.2 (2)	C10—C11—H11A	109.6
N1—C7—C6	109.10 (19)	N1—C11—H11A	109.6
C8—C7—C6	116.0 (2)	C10—C11—H11B	109.6
N1—C7—H7	107.0	N1—C11—H11B	109.6
C8—C7—H7	107.0	H11A—C11—H11B	108.1
C6—C7—H7	107.0	C11—C10—S1	109.6 (2)
C7—C8—H8A	109.5	C11—C10—H10A	109.7
C7—C8—H8B	109.5	S1—C10—H10A	109.7
H8A—C8—H8B	109.5	C11—C10—H10B	109.7
C7—C8—H8C	109.5	S1—C10—H10B	109.7
H8A—C8—H8C	109.5	H10A—C10—H10B	108.2
H8B—C8—H8C	109.5		
			
C12—N2—C9—N1	−177.6 (2)	C1—C6—C7—C8	152.3 (2)
C12—N2—C9—S1	2.1 (3)	C5—C6—C7—C8	−30.3 (3)
C10—S1—C9—N2	179.1 (3)	C9—N2—C12—N3	177 (100)
C10—S1—C9—N1	−1.1 (2)	C6—C1—C2—C3	1.1 (4)
N2—C9—N1—C11	−173.2 (2)	C5—C4—C3—C2	−0.6 (4)
S1—C9—N1—C11	7.0 (3)	C5—C4—C3—Cl1	179.5 (2)
N2—C9—N1—C7	−2.4 (4)	C1—C2—C3—C4	−0.2 (4)
S1—C9—N1—C7	177.89 (18)	C1—C2—C3—Cl1	179.7 (2)
C2—C1—C6—C5	−1.1 (4)	C3—C4—C5—C6	0.6 (4)
C2—C1—C6—C7	176.5 (2)	C1—C6—C5—C4	0.2 (4)
C9—N1—C7—C8	−98.3 (3)	C7—C6—C5—C4	−177.3 (2)
C11—N1—C7—C8	72.0 (3)	C9—N1—C11—C10	−10.7 (4)
C9—N1—C7—C6	133.2 (2)	C7—N1—C11—C10	178.5 (3)
C11—N1—C7—C6	−56.5 (3)	N1—C11—C10—S1	9.2 (4)
C1—C6—C7—N1	−82.5 (3)	C9—S1—C10—C11	−4.8 (3)
C5—C6—C7—N1	94.9 (3)		

### Hydrogen-bond geometry (Å, °)

**Table:**

*D*—H···*A*	*D*—H	H···*A*	*D*···*A*	*D*—H···*A*
C10—H10A···S1^i^	0.97	2.87	3.799 (4)	160

Symmetry codes: (i) −*x*, *y*−1/2, −*z*+3/2.
